# Redefining Cognitive Domains in the Era of ChatGPT: A Comprehensive Analysis of Artificial Intelligence’s Influence and Future Implications

**DOI:** 10.18103/mra.v12i6.5383

**Published:** 2024-06-24

**Authors:** Souvik Dubey, Ritwik Ghosh, Mahua Jana Dubey, Subhankar Chatterjee, Shambaditya Das, Julián Benito-León

**Affiliations:** 1 Department of Neuromedicine, Bangur Institute of Neurology (BIN) Kolkata, West Bengal, India; 2 Department of General Medicine, Burdwan Medical College, and Hospital, Burdwan, West Bengal, India; 3 Department of Psychiatry, Berhampore Mental Hospital, Berhampore, West Bengal, India; 4 Department of Endocrinology and Metabolism, Medical College Kolkata, Kolkata, West Bengal, India; 5 Department of Neurology, University Hospital “12 de Octubre”, Madrid, Spain; 6 Instituto de Investigación Sanitaria Hospital 12 de Octubre (imas12), Madrid, Spain; 7 Centro de Investigación Biomédica en Red Sobre Enfermedades Neurodegenerativas (CIBERNED), Madrid, Spain; 8 Department of Medicine, Faculty of Medicine, Complutense University, Madrid, Spain

**Keywords:** ChatGPT, cognition, artificial intelligence

## Abstract

**Background and Objectives:**

Despite its extensive utilization, research on Chat Generative Pre-trained Transformer (ChatGPT)’s potential negative impact on specific cognitive processes is scarce. This article explores the widespread use of ChatGPT in educational, corporate, and various other sectors, focusing on its interaction with distinct cognitive domains such as attention, executive function, language, memory, visuospatial abilities, and social cognition.

**Methods:**

A literature review was conducted using PubMed, identifying 256 articles, with 29 peer-reviewed articles analyzed after screening for relevance.

**Results:**

The review emphasizes the extraordinary capabilities of the human brain, which often go unrecognized, and argues for the importance of maintaining and enhancing natural cognitive abilities using artificial intelligence tools like ChatGPT as an aid rather than a replacement. The findings highlight the advanced reasoning capabilities of ChatGPT, blending intuitive and deliberate cognitive processes.

**Conclusion:**

Building a socio-cognitive architecture for collective human-machine intelligence has significant potential. While ChatGPT offers impressive capabilities, over-reliance on it for cognitive tasks can lead to the erosion of essential skills. It is crucial to find a balance between leveraging artificial intelligence’s advantages and preserving our natural cognitive abilities, ensuring continuous practice and engagement in traditional cognitive exercises.

## Background And Objectives

Human cognition functions are based on intricate, intriguing, interdependent, and crucially orchestrated crosstalk among subdomains of the cognitive sphere.^1^ Copacetic connections among the subdomains, i.e., attention, executive function, language, memory, visuospatial, visuoperceptual, gnosis, and praxis, build human cognition’s backbone.^2^ Human beings act as monads of society, and the aspect of human cognition dispersed socially for integration of our social attributions has been termed social cognition. Social cognition encompasses self and non-self identification, emotion perception, empathy, and theory of mind.^3,4^ Each component of social cognition entails a supreme excursion of achieved cognitive abilities in a coordinated fashion. Genetics, epigenetics, and environmental influences perpetually imbue social behavior modifications intending to balance ever-changing social norms, regulations, and customized social cognition for better and healthier social survival.

Human behavior, conditioned by an intricate interplay of personality, social cognition, learning, and past experiences, customarily steers in a patterned way determined by situational complexity. Hence, cognitive abilities, including social cognition and human behavior, have numerous determinants underneath. Each subdomain evolutional perspective and framework is further complex and undergoes multifactorial processing, which begins during infancy and then sustains several modifications throughout the lifetime by innumerable and variable sensory inputs, learning, and acquired experiences. Sensory inputs through various modalities, i.e., acquisition of information and processing of procured information, encounter thousands of modifications (i.e., trials, errors, and checkers’ balance).^5,6^ After achieving the custom-tailored information, it is further subjected to several cornerstones of cognitive abilities, i.e., learned experiences, situational analysis, concept building during the crosstalk, and the ultimate experience-dependant expression (i.e., verbal, gestural and/or by writing). The central executive network plays a role in the middle through established bidirectional connections with auditory inputs (via a phonological loop) and visuospatial inputs (via visuospatial sketchpad). This fact argues that constant multimodal sensory inputs with numerous bidirectional modifications are prerequisites for supreme executive functions. Human beings are privileged with the supreme cognitive ability of “thought,” which requires the engagement of multiple connectomes in the brain at a point in time based on attention, knowledge/concepts (i.e., semantics), learning, experiences, memory, emotions, imageries, imaginations, and anticipations. “Thought” is the cognitive ability that requires the functioning of every subdomain at a precise point in time. We “execute” our thoughts through a “thought-action” framework, which demands an even more widely and complex network of planning, organization, and sequencing in addition to “thought” and motor networks. In this way, our cognitive circuits have become increasingly strengthened and fine-tuned with errors, error checks, and corrections. Writing skill is one of the most valuable, supreme, and finest modalities to express our “thoughts” in written form. It is presumably clear from the discussion above that writing skill is a direct reflector of composite cognitive functions of the human brain, and it requires constant and regular excursions of all cognitive domains only to sharpen cognitive circuits and brain function as a whole.^7–9^

Chat Generative Pre-trained Transformer (ChatGPT) is an artificial intelligence-powered language model developed by OpenAI and released in November 2022 that can generate detailed and extensive written information virtually on any topic within seconds. Scientists have already argued whether ChatGPT can be considered an author in scientific literature. Students, teachers, corporate houses, and people from various streams have started using ChatGPT to integrate information and writing. Discussions have already gained attention on ChatGPT, and some researchers also wanted to categorize this invention as a revolution. ChatGPT provides a plethora of integrated information on any given topic within seconds.

We aimed to elucidate the interaction between ChatGPT and specific cognitive domains, including attention, executive function, language, memory, visuospatial abilities, and social cognition, and to assess the potential future impacts of ChatGPT on these cognitive processes.^10–12^

## Methods

In crafting this exploratory review paper, we conducted a targeted literature review using the PubMed database with the following keywords ([“Cognition” OR “Cognitive AND ChatGPT” OR “Chat Generative Pre-trained Transformer”]) up to May 20, 2024. We also hand‐searched additional ChatGPT-specific articles using the reference list of the selected studies and relevant journal websites from 2022 to the current date for literature inclusion. To decrease publication bias, we invigilated the references of all studies potentially missed in the electrical search. Content experts also searched the gray literature for any relevant articles.

The initial search yielded a total of 256 articles. After removing duplicates, we screened titles and abstracts for relevance, narrowing down to 29 articles for in-depth assessment. The selection process was carried out by two independent reviewers, with any discrepancies resolved through discussion or by consulting a third reviewer, ensuring a robust and unbiased selection.

This exploratory analysis enabled us to leverage a diverse array of sources to underpin our discussion and ensure a thorough examination of the presented topics.

## Results

Cultural modulation of neural mechanisms asserts that technological interactions significantly influence brain development and cognitive abilities. The use of artificial intelligence chatbots like ChatGPT for cognitive offloading may lead to underemployment of specific cognitive faculties, inhibiting their full maturation. This phenomenon is particularly relevant in the context of executive functions, where reliance on artificial intelligence for problem-solving can reduce cognitive effort and lead to long-term cognitive changes.^13^

Considering the cognitive adjustment hypotheses, three potential scenarios for the impact of pervasive ChatGPT use could be proposed: First, the cognitive zero-sum adjustment hypothesis suggests minimal interaction with neurocognitive abilities. Second, the cognitive-soft adjustment hypothesis posits that ChatGPT will be used as external resources for specific tasks, leading to potential underutilization of higher-order cognitive functions. Finally, the cognitive-hard adjustment hypothesis envisions a dystopian scenario where artificial intelligence chatbots completely replace higher-level executive functions, fundamentally altering human cognition.

There are numerous reports in the literature detailing how ChatGPT functions and what its prospects are. However, there is a notable lack of articles examining the potential adverse effects of ChatGPT on specific cognitive processes. Of the 29 articles that were ultimately selected, none systematically addressed the interaction between ChatGPT and distinct cognitive domains. Therefore, we will express our perspective on this subject, considering each primary cognitive domain, including attention, executive function, language, memory, visuospatial abilities, and social cognition.

### Attention domain:

Writing requires sustained attention, which improves with practice. In the era of ChatGPT, people have seemed to forget the degree of attention needed to construct a good write-up. The reinforcing circuit of attention involved in writing will be at stake. The primary acquisition and/or expression of any cognitive function demands attention, and anything that is expected to be related with potential to decay attention must not be welcomed, at least in every field.

### Executive function domain:

Writing is no longer considered solely a language function; it requires the engagement of executive networks. Executive functions, require the functioning of multimodal brain networks. By showing dependence on ChatGPT for every minor to significant write-up, there is a chance of losing one of the most critical executive functions (i.e., writing) soon. This executive loss will be something that will be worth seriously considering as it will be painful to see humans using ChatGPT even to resolve minor issues.

A study by Hagendorff et al.^14^ investigated how large language models like ChatGPT exhibit human-like intuitive thinking and reasoning biases. The study found that:

- Intuitive Behavior: Larger language models displayed more human-like intuitive (system 1) thinking and cognitive errors, but this decreased significantly in ChatGPT models.
- Chain-of-thought reasoning: ChatGPT models effectively use chain-of-thought reasoning to improve accuracy, mimicking human system 2 thinking.

In cognitive reflection tests and semantic illusions, both ChatGPT-3.5 and 4 models showed higher accuracy and fewer intuitive errors compared to earlier large language models^14^. These findings highlight the advanced reasoning capabilities of ChatGPT, blending intuitive and deliberate cognitive processes.

### Language domain:

Writing is the most critical aspect of the language domain within the human cognitive sphere, contributing significantly to our dominance on Earth. Writing ability depends on basic comprehension, knowledge (semantics), and grammar, making it one of the most complex language subdomains. Furthermore, writing enhances other subdomains within the language domain. However, in the era of ChatGPT, where creating a manuscript is as simple as giving a command, the pursuit of excellent writing exercises and skills is diminished. This reduction in writing practice can negatively impact other cognitive subdomains, further weakening the overall cognitive abilities.

### Memory domain:

Writing has an intriguing association with memory. In order to prepare a write-up, human beings must rely on semantics and episodic memories. Acquisition of semantics occurs through multimodal sensory inputs, and acquisition of episodic memory requires registration, encoding, rehearsals, consolidation, storage, and timely retrieval. Steps often have a bidirectional relationship with intentions to boost others and demand rehearsals and training, which means practice and exercise. Writing needs all the abovementioned components: emotion, imagination, and imagery. The authors want to express their concern that over-dependence on ChatGPT will silently snatch away the excursion of these abovementioned cognitive abilities from human beings.

### Visuospatial, visuoperceptual, praxis, and gnosis functions:

As the authors described, writing extends beyond the boundaries of the language domain. It also involves a comprehensive network of visuospatial and perceptual abilities, imagery, and imagination. These elements encompass visuospatial attention, perception, and visual memory associated with thought. “Gnosis” refers to modality-specific recognition of “what,” making it a critical component of writing. Praxis in writing is self-explanatory. Therefore, relying on a “ready-made” write-up by ChatGPT may undermine these supreme cognitive abilities, limiting room for deeper perceptions. The limitless and reckless use of ChatGPT, particularly among students, may hinder the development of these finer cognitive abilities associated with writing.

### Planning, organization, novelty, and creativity:

It is needless to mention that higher frontal lobar associates like planning, organization, sequencing, novelty seeking, and creativity are the delicate aspects that are not only essential for writing but also determine the quality of writing. The “made up” writing by ChatGPT, in all aspects, may blunt the finer tunings in the future, as these essential skills will no longer be required.

A recent study compared how humans and ChatGPT retell stories.^15^ The study highlighted several key findings:^15^

- Creativity and Novelty in Retellings: Humans create 55–60% of novel words and concepts in each iteration of retelling. ChatGPT provides competent summaries with fewer changes in subsequent retellings.
- Word Count and Part-of-Speech Differences: Both ChatGPT and humans shorten stories, but ChatGPT’s retellings are more consistent. Humans use more verbs, adverbs, and negations, while ChatGPT uses more nouns, adjectives, and prepositions.
- Concepts: Creativity, Stability, and Decay: ChatGPT uses fewer synsets initially but maintains higher synset density across retellings. Humans generate more novel synsets in later retellings.
- Age of Acquisition: Human retellings preserve words learned earlier in life, while ChatGPT increases the average age of acquisition in retellings.
- Affect Preservation: Both human and ChatGPT retellings maintain the emotional core of stories with high fidelity.

These findings reveal significant differences in language use and creativity between humans and ChatGPT in the context of retelling stories. Humans demonstrate greater variability and creativity, altering stories more significantly across iterations. They introduce new words and concepts, mainly focusing on verbs and adverbs, which reflect actions and emotions. Humans also use more negations, which suggests a nuanced understanding of context and narrative complexity.^15^

In contrast, ChatGPT’s retellings are more consistent, maintaining a higher degree of stability in word choice and structure. ChatGPT favors nouns and adjectives, focusing on the entities and descriptive aspects of the narrative. The use of later-acquired vocabulary by ChatGPT indicates a different processing mechanism compared to humans, who rely more on early-acquired, more easily retrievable words.^15^

Despite these differences, both humans and ChatGPT effectively preserve the emotional core of the stories they retell. This emotional stability suggests that while the words and specific details may change, the underlying emotional narrative remains intact, providing a consistent affective experience for the audience.^15^

### The behavior domains:

Quest and thirst for knowledge, concepts, and wisdom have made us what we boast about ourselves today. The search for knowledge should be spontaneous, open, and not limited by boundaries and ‘spoon-feeding.’ In this way, brain networks are boosted, and synaptogenesis and neuroplasticity develop. Threading scattered and fragmented knowledge and concepts in various permutations and combinations associated with novelty seeking towards more significant concepts and inventions is the supreme-most cognitive ability considered a monopoly of human beings on Earth. Most importantly, it is necessary to engage brain circuits in developing new thoughts and concepts, which depend on already achieved skills, concepts, knowledge, learning, and experiences. Authors express their deep concern that the “made” write-up by ChatGPT will undoubtedly flood the human brain with integrated information but at the cost of helpless and mindless surrendering of thought, reasoning, innovation, executive functions, imagination, novelty seeking, creativity, and writing abilities. Human brain circuits will become lazy by long-term non-utilization. They ultimately will become “sedentary-brain,” much like our current lifestyle. Researchers will need to find new ways to counteract the negative effects of a sedentary brain, including the potential rise of a new behavioral addiction: ChatGPT addiction.

#### COMPARISON OF HUMAN AND LARGE LANGUAGE MODELS IN THEORY OF MIND TASKS

This capacity to understand and interpret the mental states of others is referred to as theory of mind.^16^ Theory of mind is fundamental to human social interactions, encompassing aspects such as communication, empathy, and decision-making in social contexts.^16^ It has been a focal point of research for developmental, social, and clinical psychologists for many years. In a recent study by Strachan et al.,^17^ the performance of large language models such as GPT-4, GPT-3.5, and LLaMA2-70B was compared with human participants across various theory of mind tasks. The tasks included understanding false beliefs, interpreting indirect requests, recognizing irony and faux pas, and understanding complex mental states.^17^

- False Belief Task: Both human participants and large language models performed at ceiling levels in the false belief task, demonstrating similar capabilities in understanding where an agent would look for an object based on their memory rather than its current location.
- Irony Task: GPT-4 outperformed humans in recognizing ironic statements, while GPT-3.5 and LLaMA2-70B performed below human levels.
- Faux Pas Task: GPT-4 and GPT-3.5 struggled with detecting faux pas, whereas LLaMA2-70B performed at or above human levels. However, further analysis suggested that the superior performance of LLaMA2-70B might be due to a bias towards attributing ignorance.
- Hinting Task: GPT-4 again showed superior performance compared to humans, while GPT-3.5 was comparable to human performance, and LLaMA2-70B performed worse.
- Strange Stories Task: GPT-4 significantly outperformed humans, whereas GPT-3.5 was on par with humans, and LLaMA2-70B scored lower.

These findings highlight that while large language models like GPT-4 exhibit advanced capabilities in some aspects of the theory of mind, they also have specific areas where their performance diverges from human-like understanding, particularly in tasks requiring nuanced social inferences.^17^ The implications of these results are significant for understanding the potential and limitations of large language models in replicating human cognitive processes. The varying performance across different tasks suggests that while large language models can excel in certain cognitive functions, they may still rely on different mechanisms compared to humans.

## Conclusion

The human brain functions in ways that often go beyond our conscious perception, leaving much of its true potential unacknowledged. Regular activities such as reading and writing play a crucial role in activating numerous brain circuits, thereby enhancing integrated cognitive functions. These activities engage various cognitive domains, including attention, executive function, language, memory, visuospatial abilities, and social cognition.

While artificial intelligence chatbots like ChatGPT offer significant advantages as cognitive enhancers, their impact on long-term cognitive development must be carefully considered. Understanding and anticipating these effects will allow educational and political communities to formulate strategies to mitigate potential adverse outcomes and enhance the positive aspects of artificial intelligence integration into daily life. With the widespread use of ChatGPT, it is essential to recognize the value of maintaining traditional cognitive exercises. Writing, for instance, is not just a language skill but a complex cognitive activity that involves comprehension, knowledge, grammar, visuospatial and perceptual abilities, and imaginative thinking. Engaging in the act of writing helps strengthen these cognitive domains and promotes overall brain health and functionality.

González et al.^18^ emphasizes the potential of building a socio-cognitive architecture for collective human–machine intelligence. This architecture involves planning and coordinating interdependent actions, integrating individual cognitive and metacognitive capacities to achieve common goals. Such integration can enhance collaborative efforts, providing significant societal benefits, including improved decision-making, innovative solutions to complex problems, and enhanced overall productivity.

While ChatGPT offers impressive capabilities and can serve as a valuable tool in various contexts, over-reliance on it for cognitive tasks can lead to the erosion of these essential skills. The ease of generating ready-made content might discourage the practice of writing and critical thinking, leading to potential declines in cognitive abilities. This is particularly concerning for students and individuals in learning environments where the development of these skills is paramount.

We must strive to find a balance between leveraging the advantages of artificial intelligence and preserving our natural cognitive abilities. By continuing to engage in activities that challenge and stimulate our brains, we can ensure that we do not lose the finely tuned cognitive skills that have been developed through centuries of human evolution. Ultimately, while artificial intelligence can aid and enhance our capabilities, it is crucial to remember that the supreme cognitive functions endowed by Nature require continuous practice and engagement. We should embrace artificial intelligence as a tool to complement and support our cognitive endeavors, not as a replacement for the inherent abilities that define human intelligence.

## Data Availability

The data generated or analyzed during this study are available from the corresponding author upon reasonable request.
